# Detection of cellular prion protein in exosomes derived from ovine plasma

**DOI:** 10.1099/jgv.0.000291

**Published:** 2015-12

**Authors:** Elena Berrone, Cristiano Corona, Maria Mazza, Elena Vallino Costassa, Monica Lo Faro, Francesca Properzi, Chiara Guglielmetti, Cristiana Maurella, Maria Caramelli, Maria Chiara Deregibus, Giovanni Camussi, Cristina Casalone

**Affiliations:** ^1^​ Istituto Zooprofilattico Sperimentale del Piemonte, Liguria e Valle d'Aosta, Via Bologna 148, 10154 Turin, Italy; ^2^​ Department of Cellular Biology and Neuroscience, Istituto Superiore di Sanità, Viale Regina Elena 299, 00161 Rome, Italy; ^3^​ Department of Internal Medicine and Molecular Biotechnology Center, University of Turin, Corso Dogliotti 14, 10126 Turin, Italy

## Abstract

Prion protein (PrP) is present at extremely low levels in the blood of animals and its detection is complicated by the poor sensitivity of current standard methodologies. Interesting results have been obtained with recent advanced technologies that are able to detect minute amounts of the pathological PrP (PrP^Sc^), but their efficiency is reduced by various factors present in blood. In this study, we were able to extract cellular PrP (PrP^C^) from plasma-derived exosomes by a simple, fast method without the use of differential ultracentrifugation and to visualize it by Western blotting, reducing the presence of most plasma proteins. This result confirms that blood is capable of releasing PrP in association with exosomes and could be useful to better study its role in the pathogenesis of transmissible spongiform encephalopathies.

Transmissible spongiform encephalopathies (TSEs) are a family of rare progressive neurodegenerative disorders characterized by abnormal brain deposition of an insoluble and protease-resistant isoform of cellular prion protein (PrP^C^) named PrP^Sc^. Invariably fatal, TSEs include a wide range of animal and human conditions of sporadic, genetic or infectious origin, such as bovine spongiform encephalopathy in cattle, scrapie in sheep and goats, chronic wasting disease in deer and elk, and Creutzfeldt–Jakob disease in humans.

In recent years, it has been demonstrated that PrP is associated with exosomes, small membrane vesicles of endocytic origin which play an important role in intercellular communication. PrP is associated with exosomes secreted from non-neuronal and neuronal cells (Fevrier *et al.*, 2004; Leblanc *et al.*, 2006; Wang *et al.*, 2011), and exosomes containing PrP^Sc^ are capable of transferring infectivity to cells (Vella *et al.*, 2007; Alais *et al.*, 2008). Despite the unequivocal presence of blood infectivity of prion-affected individuals, PrP detection might be masked by proteins or soluble components of plasma (Abdel-Haq, 2015; Gregori *et al.*, 2008; Orrú *et al.*, 2011; Properzi *et al.*, 2015; Saá *et al.*, 2014).

In order to study the role of plasma-derived exosomes in the pathogenesis of TSEs and their potential as a tool for *in vivo* diagnosis, we devised a simple method to significantly enrich plasma-derived PrP after exosome extraction in blood. To do this, we used plasma samples (Table S1, available in the online Supplementary Material) from five sheep naturally infected with scrapie, confirmed PrP^Sc^-positive by confirmatory tests (Mazza *et al.*, 2010), and three healthy sheep, selected from livestock with no record of scrapie cases in the past 5 years and selected for scrapie resistance by genetic analysis (Hunter, 2007).

A crucial step in exosome preparations is to obtain highly purified vesicles as plasma is a complex and viscous fluid with a high protein concentration (60–80 mg ml^ − 1^) (Momen-Heravi *et al.*, 2012; Salazar Vázquez *et al.*, 2009). The most commonly used method to isolate and purify exosomes is by differential centrifugation coupled with ultracentrifugation (Welton *et al.*, 2010). However, some contaminants such as protein complexes and co-sedimenting vesicles might compact with exosomes when using this method (Tauro *et al.*, 2012). In our modified precipitation method, 1 ml each plasma sample was incubated with 252 μl polymeric precipitation mixture according to the manufacturer's instructions (Fresenius Medical Care) at 4 °C overnight and then centrifuged at 1500 ***g*** for 30 min at 4 °C. The exosome pellets were lysed using 500 μl RIPA buffer (Sigma-Aldrich) and protease inhibitors were added (Pefabloc SC Plus; Roche). Protein quantification was performed using a BCA (bicinchoninic acid) Protein Assay kit (Thermo Scientific). This method of precipitation was compared with a classical differential ultracentrifugation protocol (10 000 ***g*** for 30 min followed by 100 000 ***g*** for 1 h) by nanoparticle tracking analysis. In particular, size and distribution of exosomes were evaluated by a NanoSight LM10 instrument (NanoSight) equipped with nta 2.0 analytic software (Dragovic *et al.*, 2011). The NanoSight analysis showed that the profile, size and number of particles isolated by the two methods were comparable (Fig. S1a, b). These results demonstrate that the precipitation method did not affect the size and concentration of plasma particles. In addition, to confirm the vesicular nature of the plasma-derived exosomes, Western blotting analysis of flotillin-1 expression, a typical marker of exosomes (Vella *et al.*, 2008a, b), was performed. As compared with tissue lysate [brain homogenate (BH)], extracted as described below, flotillin-1 was found to be enriched in exosome lysate (Fig. 1). Flottilin-1 is a constituent of lipid subdomains in recycling exosomes and is normally more enriched in exosome lysate than in cell/tissue lysate (Grey *et al.*, 2015; Meister & Tikkanen, 2014; Vella *et al.*, 2008a, b).

**Fig. 1.**
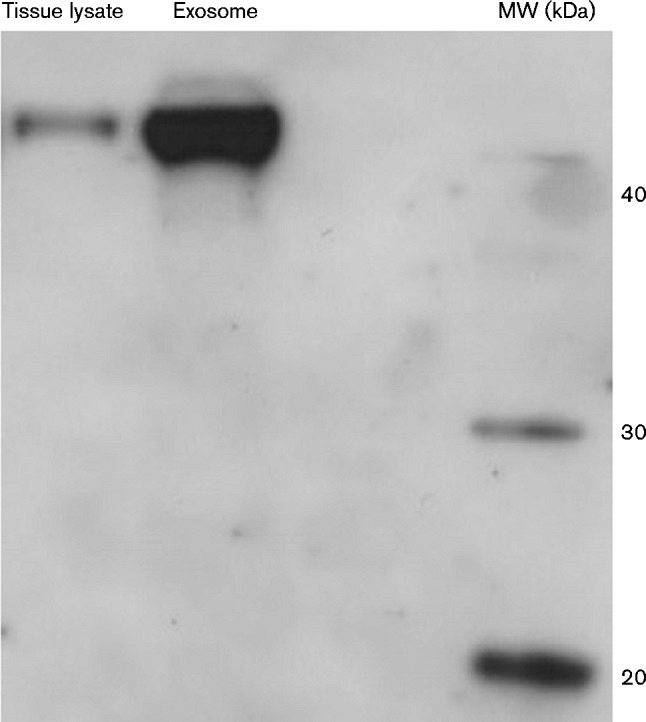
Expression of the plasma exosome marker flotillin-1 by Western blotting. Equivalent amounts (125 μg total protein) of scrapie-infected sheep BH (Tissue lysate) and exosome lysate (Exosome) were analysed by Western blotting using an anti-flotillin-1 (45 kDa) antibody (BD Biosciences). MW, molecular mass markers.

We next assessed the expression of PrP from exosome lysates by Western blotting using the anti-PrP mAb P4 (R-Biopharm), routinely used due to its high sensitivity and specificity in confirmatory Western blotting assays for active scrapie outbreak surveillance, as described previously (Arsac *et al.*, 2007; Baron *et al.*, 2007; Harmeyer *et al.*, 1998; Mazza *et al.*, 2010). However, the presence of plasma protein bands did not allow the identification of the typical PrP three-band Western blotting pattern (Raymond & Chabry, 2004) (Fig. S2), as reported previously (Gregori *et al.*, 2008; Properzi *et al.*, 2015; Saá *et al.*, 2014).

To improve PrP visualization, we performed a spike-in experiment where a known protein amount of sheep BH was added to the plasma from healthy animals. This experiment was done to test a method for PrP detection in dilute fluids such as plasma, but using samples effectively containing PrP^C/Sc^. The brains were homogenized in 10 % sarcosyl and clarified by ultracentrifugation as described previously (Bozzetta *et al.*, 2004). After protein quantification, dilutions of total BH protein from scrapie-positive (SBH) and/or scrapie-negative (NBH) sheep were spiked into 500 μl healthy sheep plasma to give the following dilutions: (1) 500 pg NBH μl^ − 1^ (100% negative), (2) 250 pg NBH μl^ − 1^ (50% negative)+250 pg SBH μl^ − 1^ (50% positive) and (3) 500 pg SBH μl^ − ^ (100% positive).

To concentrate the PrP protein, 40 μl of all spiked plasma samples were precipitated with thyroglobulin (TG; 5 mg ml^ − 1^), a large unrelated soluble protein used as a carrier in protein precipitation methodologies (Kocisko *et al.*, 1995), and 4 vols methanol at  − 20 °C for 2 h, and then centrifuged. The spike-in samples, positive BH (C+) and negative BH (C − ) were analysed to detect PrP using the mAb P4 (Harmeyer *et al.*, 1998) (Fig. 2a, b). To demonstrate the specificity of P4 and the possible interaction of secondary antibody, the samples were examined using only non-immune IgG as control (Fig. 2c, d). In no-proteinase K (PK) ( − )- and PK (+)-digested samples it was possible to compare the different PrP glycoform patterns (Raymond & Chabry, 2004): PrP^C^ showed the three bands (di-, mono- and unglycosylated) between 20 and 37 kDa, and PrP^Sc^ between 17 and 29 kDa (Fig. 2a, b).

**Fig. 2.**
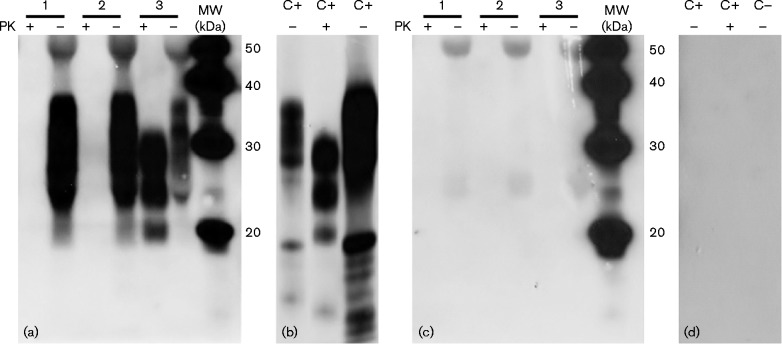
Detection of ovine brain-derived PrP spiked into healthy ovine plasma after TG/methanol precipitation. PK (+)- and no-PK ( − )-digested samples were analysed by Western blotting using the anti-PrP mAb P4. (a) Lane 1, 500 pg total NBH protein μl^− 1^ (100% negative); lane 2, 250 pg total NBH protein μl^− 1^ (50% negative)+250 pg total SBH protein μl^− 1^ (50% positive); lane 3, 500 pg total SBH protein μl^− 1^ (100% positive). (b) C+, scrapie-infected sheep BH (positive control); C − , healthy sheep BH (negative control). (c, d) Non-immune IgG Western blotting of the same samples. Images were acquired with two different exposure times: (a, c) 10 min and (b, d) 3 min. With regard to the PrP patterns: no-PK lane 1 ( − ) is similar to no-PK ( − ) C − , PK (+) lane 1 is negative; no-PK ( − ) lane 2 is similar to no-PK ( − ) C − , PK (+) lane 2 is negative; no-PK ( − ) lane 3 is similar to no-PK ( − ) C+, PK (+) lane 3 is similar to PK (+) C+. MW, molecular mass markers.

To verify the reduction of total plasma/exosome protein during the PrP isolation, silver staining after PAGE was performed. It was confirmed that the greater concentration of total protein was present in undiluted plasma, whereas exosome lysate, after TG/methanol precipitation, showed an evident reduction of total protein (Fig. S3).

As reported in other similar studies with blood samples (Bannach *et al.*, 2012; Bellon *et al.*, 2003; Caplazi *et al.*, 2004; Gregori *et al.*, 2008; Lau *et al.*, 2007; Saá *et al.*, 2014), different approaches can be used to concentrate PrP and spike-in experiments utilized to verify method sensitivity. In our study, our precipitation method proved useful to reduce the highly unspecific signal background and the spike-in experiment allowed us to determine the limit of detection (500 pg total BH protein μl^ − 1^) in order to visualize PrP^C/Sc^ by Western blotting.

To detect PrP in the exosomes, the same protocol described above was used on exosomes derived from eight plasma samples of scrapie-infected or healthy sheep: 40 μl exosome lysate was precipitated with TG and 4 vols methanol at  − 20 °C for 2 h and then centrifuged. The pellets were dissolved in Laemmli buffer and an equal volume (20 μl) was loaded for Western blotting. Although the heavy chain at 55 kDa and the light chain at 24 kDa of IgG were visible, it was possible to observe the diglycosylated band at ∼37 kDa in all eight no-PK-digested samples and the monoglycosylated band at ∼30 kDa in four samples (E3, E5, E7 and E8) (Fig. 3a). To confirm these results, the signal specificity was detected as described earlier (Fig. 3c). The different visualization of the PrP^C^ bands could have been due to different protein concentrations of each sample; in fact, band intensity was more evident in the samples with a higher amount of protein (Table S1). In order to detect PrP^Sc^, the same samples were PK-digested and loaded in a separate gel. Although the unspecific bands of IgG disappeared, it was not possible to visualize the PrP^Sc^ pattern bands (Fig. S4).

**Fig. 3.**
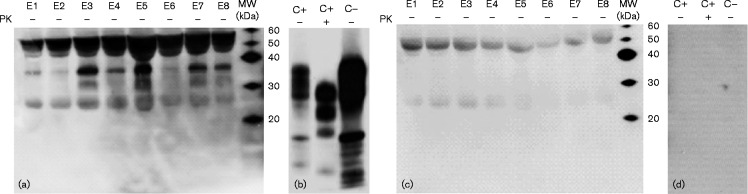
PrP^C^ in ovine plasma-derived exosomes after TG/methanol precipitation. Using Western blotting, an equal volume (20 μl) of samples was analysed for PrP^C^ expression. (a) Eight no-PK ( − )-digested exosome lysates: five sheep naturally infected with scrapie (E1, E2, E3, E6 and E7) and three healthy sheep (E4, E5 and E8). (b) C+, PK (+)- and no-PK ( − )-digested scrapie-infected sheep BH (positive control); C − , no-PK ( − )-digested healthy sheep BH (negative control). (c, d) Non-immune IgG Western blotting of the same samples. Images were acquired with two different exposure times: (a, c) 40 min and (b, d) 3 min. MW, molecular mass markers.

The novelty of our study resides in the demonstration that PrP^C^ is indeed associated with exosomes isolated from sheep plasma and can be visualized by Western blotting.

Low amounts of PrP^C/Sc^ are present in a variety of peripheral tissues and body fluids (Franscini *et al.*, 2006; Franz *et al.*, 2012; Hampton, 2006; Henderson *et al.*, 2015; Mulcahy *et al.*, 2004; Murayama *et al.*, 2014; WHO, 2010), but it is difficult to detect with current biochemical and immunohistochemical assays. Recently, it was possible to detect PrP^TSE^ using an immunochemistry technique in plasma exosomes of prion-infected rodents; however, a pool of 5 ml hamster blood and a sucrose gradient by ultracentrifugation were needed in order to visualize the PrP^C/Sc^ (Properzi *et al.*, 2015). Our results indicate, instead, that it is possible to extract PrP from 1 ml plasma by a simple, fast method without the use of differential ultracentrifugation. In detail, we were able to extract a sufficient amount of purified exosomes by polymeric precipitation, with good quality total exosome protein, and TG/methanol precipitation permitted the elimination of most plasma protein contaminants that can interfere with PrP isolation.

Our results confirm that PrP^C^ associated with exosomes might contribute to the pathogenesis and transmission of prion diseases. Targeting of exosomes containing PrP^C^ could confer susceptibility to cells that do not express PrP and facilitate prion propagation (Porto-Carreiro *et al.*, 2005). Moreover, cellular levels of PrP^C^ are known to regulate the amount of secreted vesicles with a major role in health and disease (Dias *et al.*, 2015). Further studies are needed to elucidate the intracellular trafficking route of glycosylphosphatidylinositol-anchored proteins, such as PrP, from the caveolar pathway to extracellular vesicles. From this point of view, our simple method to detect PrP^C^-associated exosomes *in vivo* could be useful to better study the role of PrP^C^ in TSEs pathogenesis.

Further experiments combining our protocol as a (semi)purification step with other approaches could be useful to improve the detection of PrP^Sc^ in the blood of naturally TSE-infected animals. For example, good results have been demonstrated by recent PrP^Sc^ amplification technologies, such as real-time quaking-induced conversion (RT-QuIC) or protein misfolding cyclic amplification (PMCA) (Henderson *et al.*, 2015; Orrú *et al.*, 2014; Properzi & Pocchiari, 2013). PMCA was able to demonstrate the presence of PrP^TSE^ in extracellular vesicles from the plasma of mice experimentally infected with mouse-adapted variant Creutzfeldt–Jakob disease (Saá *et al.*, 2014).

Currently, most prion studies use experimentally infected animals. However, with our simple and fast approach, *in vivo* studies on naturally infected animals could provide novel insights into TSEs pathogenesis.

## Supplementary Data

1290 Supplementary Data
