# Supplementary material for: Detection of cellular prion protein in exosomes derived from ovine plasma
Source: J Gen Virol. 2015 Dec;96(Pt 12):3698–702. doi: 10.1099/jgv.0.000291 (PMC4804764; doi:10.1099/jgv.0.000291)
Supplement: Supplementary file 1 — Supplementary Data [file jgv000291.pdf]

**Supplementary material**

**Figure S1. Characterization of plasma exosomes by Nanosight**. Representative image of NanoSight analysis of exosomes purified by ultracentrifugation (a) and by polymeric precipitation mixture (b). The peaks show the relationship between particle distribution (left Y axis) and particle size (X axis); the curve describes the correlation between cumulative percentage distribution of particles (percentile in right Y axis) and particle size (X axis). Mean of size and particle concentration values were calculated by the Nanoparticle Tracking Analysis (NTA) software. This program analyzes video images of the particle's movement under Brownian motion captured by Nanosight LM10 and evaluates the diffusion coefficient, sphere equivalent and hydrodynamic radius of particles by using the Strokes-Einstein equation.

**Fig. S1**

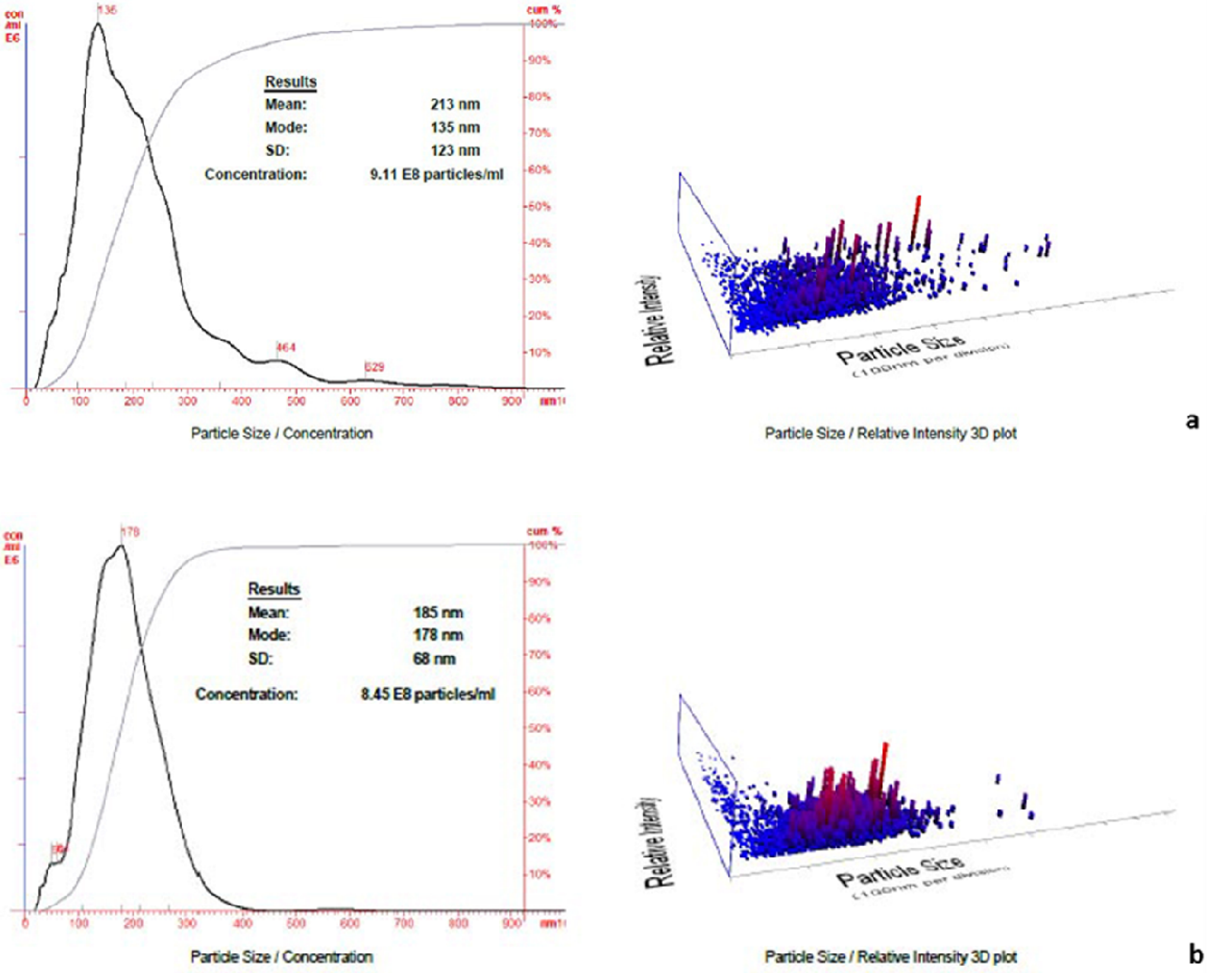
Results
Mean: 213 nm
Mode: 135 nm
SD: 123 nm
Concentration: 9.11 E8 particles/ml
136
464
629
cum %
Particle Size / Concentration
Relative Intensity
Particle Size
Particle Size / Relative Intensity 3D plot
a
Results
Mean: 185 nm
Mode: 178 nm
SD: 68 nm
Concentration: 8.45 E8 particles/ml
178
cum %
Particle Size / Concentration
Relative Intensity
Particle Size
Particle Size / Relative Intensity 3D plot
b

**Figure S2. PrP$^C$ in ovine plasma-derived exosome without TG/methanol precipitation**. 125 µg of total exosome lysate protein were analyzed by Western Blotting using the anti-PrP monoclonal antibody P4 (R-Biopharm AG).

## Fig. S2

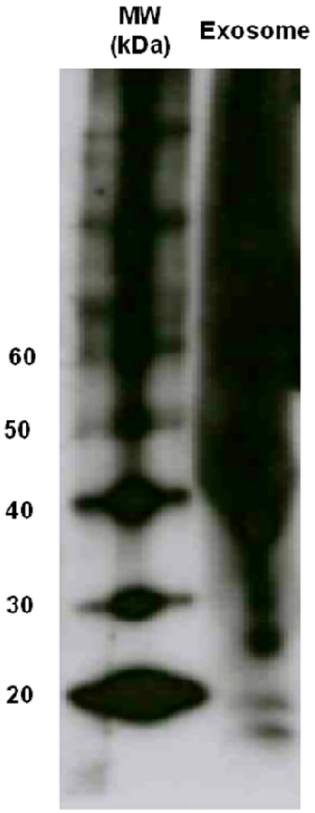

**Figure S3. Silver staining of ovine undiluted plasma and plasma-derived exosome.** After protein quantification, samples were analyzed by silver stain SDS-PAGE gel. Lane 1: 125 µg of total undiluted plasma protein; Lane 2: 125 µg of total exosome lysate protein; Lane 3: exosome lysate after TG/methanol precipitation as described in main text; Lane C+: 125 µg of PK (+) and no-PK **(-)** digested scrapie-infected sheep BH.

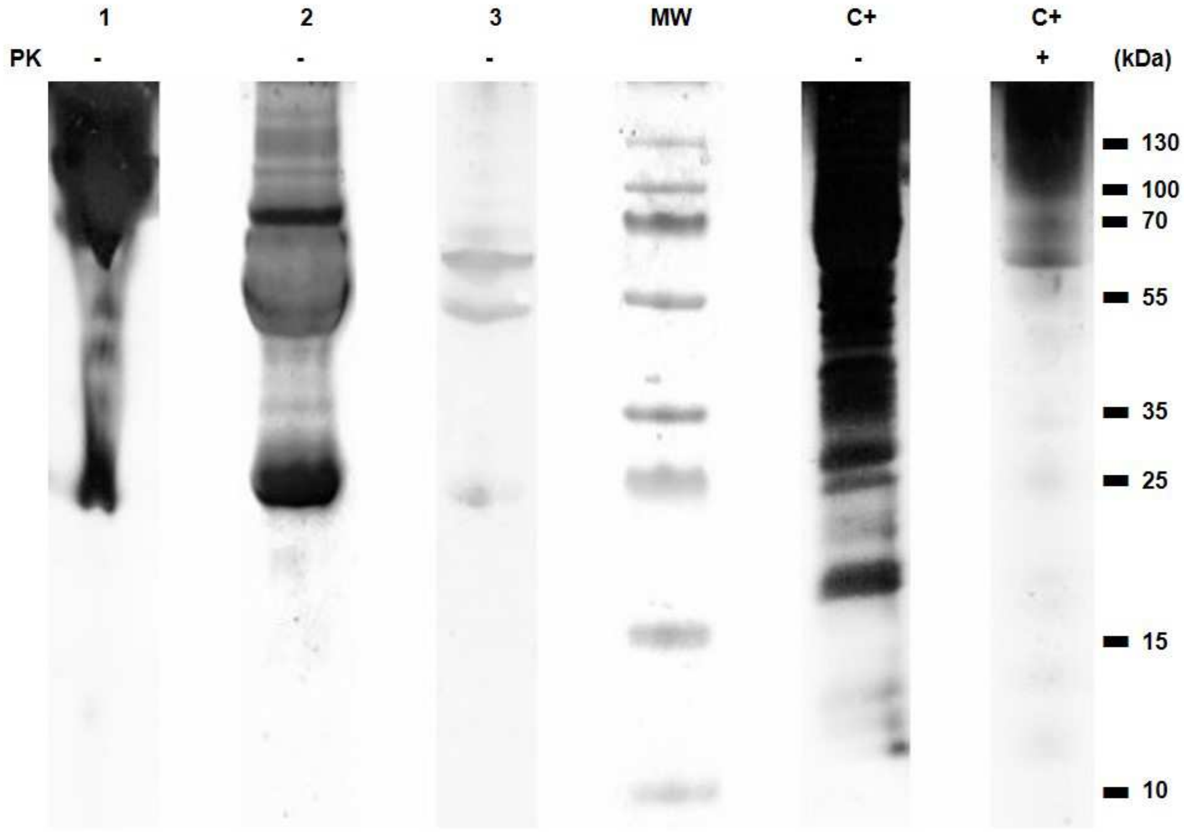

**Figure S4. $PrP^{Sc}$ in ovine plasma-derived exosome after TG/methanol precipitation.** By Western blotting, an equal volume (20 μl) of PK (+) digested samples was analyzed for PrP expression. Panel a) 8 samples of exosome lysate: 5 sheep naturally infected with scrapie (E1, E2, E3, E6, E7); 3 healthy sheep (E4, E5, E8). Panel b) C+: scrapie-infected sheep BH (positive control). Panels a 40 minutes, Panels b 3 minutes.

# Fig. S4

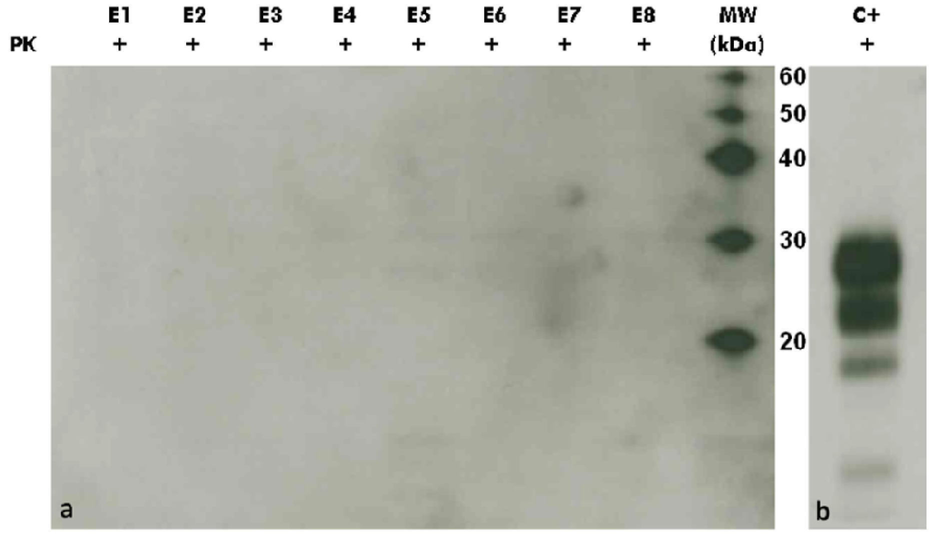

**Table S1 – Exosome protein concentrations.** Exosome lysate was obtained as described in main text from plasma of 5 sheep naturally infected with scrapie confirmed $PrP^{Sc}$ positive (+) and 3 healthy sheep (-). Protein quantification was performed by BCA Protein Assay kit (Pierce - Thermo Fisher Scientific).

| Sample ID | $PrP^{Sc}$ | Protein concn (µg/µl) |
|---|---|---|
| **E1** | + | 4,67 |
| **E2** | + | 4,95 |
| **E3** | + | 29,21 |
| **E4** | - | 8,28 |
| **E5** | - | 23,51 |
| **E6** | + | 8,82 |
| **E7** | + | 9,64 |
| **E8** | - | 9,00 |
